# Molecular identification and functional characterization of IRF4 from common carp (*Cyprinus carpio*. L) in immune response: a negative regulator in the IFN and NF-κB signalling pathways

**DOI:** 10.1186/s12917-022-03205-8

**Published:** 2022-03-17

**Authors:** Yaoyao Zhu, Guiwen Yang

**Affiliations:** 1 Key Laboratory of Tropical Marine Fishery Resources Protection and Utilization of Hainan Province, College of Fisheries and Life Science, Hainan Tropical Ocean University, No. 1 Yucai Road, Sanya, 572022 China; 2 Hainan Key Laboratory for Conservation and Utilization of Tropical Marine Fishery Resources, Hainan Tropical Ocean University, Sanya, 572022 China; 3 Shandong Provincial Key Laboratory of Animal Resistance Biology, College of Life Sciences, Shandong Normal University, No. 88 East Wenhua Road, Jinan, 250014 China

**Keywords:** Common carp (*Cyprinus carpio*. L), Interferon regulatory factor 4 (IRF4), Poly (I:C), *Aeromonas hydrophila*, Signalling pathway

## Abstract

**Background:**

The interferon (IFN) regulatory factors (IRFs) were originally identified as transcription factors playing critical roles in the regulation of IFN-related genes in the signal pathway. In mammals, IRF4 plays a vital role in both the innate and adaptive immune system. This study aims to reveal the molecular characterization, phylogenetic analysis, expression profiles and the regulatory role in the IFN and NF-κB signalling pathways of IRF4 in common carp (*Cyprinus carpio*. L) (abbreviation, ccIRF4).

**Results:**

Here, ccIRF4 was identified and characterized, it contained a DNA binding domain (DBD) which possess five tryptophans and an IRF-associated domain (IAD). The predicted protein sequence of the ccIRF4 showed higher identities with grass carp (*Ctenopharyngodon idella*) and zebrafish (*Danio rerio*). Phylogenetic analysis suggested that ccIRF4 has the closest relationship with zebrafish IRF4. Quantitative real-time PCR analysis showed that ccIRF4 was constitutively expressed in all investigated tissues with the highest expression level in the gonad. Polyinosinic:polycytidylic acid (poly I:C) stimulation up-regulated the ccIRF4 expressions in the liver, spleen, head kidney, skin, foregut and hindgut. Upon *Aeromonas hydrophila* injection, the expression level of ccIRF4 was up-regulated in all tissues with the exception of spleen. In addition, ccIRF4 was induced by lipopolysaccharide (LPS), peptidoglycan (PGN) and Flagellin in head kidney leukocytes (HKLs). Overexpression of the ccIRF4 gene in epithelioma papulosum cyprini cells (EPC) down regulated the expressions of IFN-related genes and proinflammatory factors. Dual-luciferase reporter assay revealed that ccIRF4 decreased the activation of NF-κB through MyD88.

**Conclusions:**

These results indicate that ccIRF4 participates in both antiviral and antibacterial immune response and negatively regulates the IFN and NF-κB response. Overall, our study on ccIRF4 provides more new insights into the innate immune system of common carp as well as a theoretical basis for investigating the pathogenesis and prevention of fish disease.

## Background

The innate immunity is the only defense mechanism among invertebrates, and a fundamental defense mechanism in vertebrate animals as well, especially in lower vertebrates, such as fishes, whose adaptive immunity is relatively less developed [1]. Interferon (IFN) regulatory factors (IRFs) were originally identified in the 1980s, as the transcriptional regulatory factors play important roles in regulating the expression of type I IFN and IFN-stimulated genes (ISGs) in the related signalling pathways [2]. They play multifuctions in the antiviral defense, innate and adaptive immunity, cell growth regulation and apoptosis [3]. To date, a total of 11 members have so far been reported in vertebrates, IRF10 was found absent in humans and mice, whereas IRF11 has only been identified in fish. Based on the C-terminal region and molecular phylogenetic analyses, IRF proteins can be divided into four subfamilies: IRF1 (IRF1, 2, and 11), IRF3 (IRF3 and 7), IRF4 (IRF4, 8, 9, and 10) and IRF5 (IRF5 and 6) subfamilies [4]. Each IRF has distinct functional roles, transcriptional activators (IRF1, IRF3, IRF7 and IRF9) and bi-functional factors that both activate and repress transcription depending on the target gene (IRF2, IRF4, IRF5 and IRF8) [5]. All IRF family members share a highly conserved DNA-binding domain (DBD) of approximately 115 amino acids which contain five or six tryptophan repeats at the N-terminal region, that binding to the core recognition sequence, A/GNGAAANNGAAACT, termed the IFN stimulated response element (ISRE). The C-terminal region of IRFs generally possesses the IRF associated domain (IAD), with the exception of IRF1 and IRF2 which possess a very similar IAD2. The IAD interacts with other proteins to form homo- or hetero-dimers which are required for accurate promoter targeting and regulation of transcription [6].

IRF4 (also known as MUM1, PIP, LSIRF or LCSAT) plays multifunction in the innate and adaptive immunity [7]. In mammals, IRF4 is found to be expressed in various immune cells, including T and B cells, macrophages, and dendritic cells and plays crucial roles in their development and differentiation, but its function in the myeloid lineage is not well characterized [8, 9]. IRF4 mediates immune response by activating the expression of other genes during cell differentiation [10, 11]. Regulation of cell development processes include B cell receptor editing, Ig class switching, plasma cell generation, the germinal centre reaction and Th1/Th2 immune responses [12–16]. Besides, IRF4 is a key molecule for interleukin (IL)-17 production induced by IL-21 and the development and stability of the Th17 phenotype mediated by IL-6/TGFβ [17, 18]. However, IRF4 can compete with IRF5 for Myd88 interaction and act as a negative transcription factor of Toll-like receptor (TLR) signalling [19]. In the IRF4 deficient peritoneal macrophages from mice, production of the TLR-dependent proinflammatory cytokines is significantly enhanced [19]. IRF4 negatively regulate the expression of IRF5 that overexpression of IRF4 inhibits IRF5 expression whereas IRF4 knockdown increases IRF5 expression. Moreover, IRF4 binds to IRF5 promoters and negatively regulates IRF5 promoter reporter activities [20]. In mammals, IRF1, IRF5, IRF7, IRF8 and IRF9 can be induced by poly I:C except IRF4 [21]. However, teleost IRF4 can be induced by virus, bacteria or pathogen-associated molecular patterns (PAMPs, such as poly I:C or LPS) and it has been identified in mandarin fish (*Siniperca chuatsi*) [22], Asian swamp eel (*Monopterus albus*) [23], orange-spotted grouper (*Epinephelus coioides*) [24], rainbow trout (*Oncorhynchus mykiss*) [25], Japanese flounder (*Paralichthys olivaceus*) [26], large yellow croaker (*Larimichthys crocea*) [27], turbot (*Scophthalmus maximus*) [28], rock bream (*Oplegnathus fasciatus*) [29], half-smooth tongue sole (*Cynoglossus semilaevis*) [30], zebrafish (*Denio rerio*) [31], Atlantic salmon (*Salmo salar*) [32], channel catfish (*Ictalurus punctatus*) [33], blunt snout bream (*Megalobrama amblycephala*) [34] and miiuy croaker (*Miichthys miiuy*) (unpublished data). Almost all of these studies have concentrated on the structural characteristics and expression analysis of IRF4 in fishes. However, the functional roles of fish IRF4 remain largely unknown. The characterization of IRF4 in more vertebrates will certainly enable the understanding of its evolution and its immune function.

Common carp (*Cyprinus carpio*. L) is one of the most important aquaculture species in China, as well as in other Asia and European countries. Up to now, IRF1, IRF2, IRF3, IRF5, IRF7, IRF9 and IRF10 were reported in common carp [35–40]. In this study, we aimed to determine the protein structure and function of IRF4 in common carp (named ccIRF4). We identified the full-length cDNA sequencing and characterization of ccIRF4. Apart from investigating their tissue distribution in healthy common carp, we also evaluated the responsiveness of ccIRF4 upon viral or bacterial stimulation both *in vivo* and *in vitro*. Furthermore, we determined the regulatory role of ccIRF4 in the IFN and NF-κB signalling pathways. These results will contribute to the understanding of fish innate immune response against pathogens.

## Materials and methods

### Fish and cell lines

Healthy common carp specimens (approximately 200 g per fish) were purchased from a local fish farm and cultured in recirculating tap water at 25 °C. Fish were fed daily to satiation with commercial fish feed for more than one week prior to experimental use.

Epithelioma papulosum cyprini (EPC) cells and 293 T cells were stored in our laboratory. EPC cells were maintained at 25 °C in M199 (HyClone) supplemented with 10% fetal bovine serum (FBS, Gibco), 100 U/ml penicillin (Gibco) and 100 mg/ml streptomycin (Gibco). 293 T cells were maintained at 37 °C, 5.0% CO_2_ in DMEM (HyClone) supplemented with 10% FBS, 100 U/ml penicillin (Gibco) and 100 mg/ml streptomycin (Gibco). Transfection was performed according to a previous report [41].

### Molecular cloning and sequencing of the ccIRF4

Partial cDNA sequence of ccIRF4 was obtained using a pair of degenerate primers IRF4-F/IRF4-R (Table 1) designed on the basis of the known IRF4 sequences download from NCBI data base. Then, the full-length cDNA of ccIRF4 was obtained by RACE (rapid amplification of the cDNA ends) method using 3′-Full RACE and 5′- Full RACE Core Set Kit (TaKaRa).

**Table 1:** Primers used in this study

Primer	Sequence(5′-3′)	Application
ccIRF4-F	GGAGCCAGCTGGACATCTC	Cloning for IRF4
ccIRF4-R	CAGGAGCTGCCTGGCGAAC	Cloning for IRF4
ccIRF4-5Rout	CGTCATCTGAGGCTGTAGAGGAGG	Cloning for IRF4
ccIRF4-5Rin	GAGCCTCTCTTGGCTCCTTCTGG	Cloning for IRF4
ccIRF4-3Fout	GGACACGCAGCAGTTCCTCTCAG	Cloning for IRF4
ccIRF4-3Fin	GCCACGCTCTCAGGTGGTGCTGTG	Cloning for IRF4
ccIRF4-Frt	CCAATATGAGATCCGCCGAAGCC	Rea-ltime PCR
ccIRF4-Rrt	CCTGGAGACGAAGAGGAGGAGATG	Rea-ltime PCR
ccS11-F	CCGTGGGTGACATCGTTACA	Rea-ltime PCR
ccS11-R	TCAGGACATTGAACCTCACTGTCT	Rea-ltime PCR
ccIRF4-F-EI	CCGGAATTCATGAACTTAGATGGGGACAGCAGC	Recombinant plasmid
ccIRF4-R-SII	TCCCCGCGGCACCTGCAAGTGCTGGATGCT	Recombinant plasmid
EPC-IFN-F	CGCTAAGGTGGAGGACCAGGTTA	Rea-ltime PCR
EPC-IFN-R	TTAGGTTCCATTGTGCTGCGTTCA	Rea-ltime PCR
EPC-viperin-F	AAGACTTCCTGGACCGCCATAAGA	Rea-ltime PCR
EPC-viperin-R	CCTCTCGGCAATCCAAGAAGCG	Rea-ltime PCR
EPC-PKR-F	TGGAGACTTCGGCCTCGTGACT	Rea-ltime PCR
EPC-PKR-R	TCGCTTGCTCCGGGCTCATGTA	Rea-ltime PCR
EPC-IL-1β-F	CCCAGACCAATCTCTACCTCGCT	Rea-ltime PCR
EPC-IL-1β-R	GAGGAGGTTGTCATTCTGGTCACC	Rea-ltime PCR
EPC-β-actin-F	GCCGTGACCTGACTGACTACCT	Rea-ltime PCR
EPC-β-actin-R	GCCACATAGCAGAGCTTCTCCTTG	Rea-ltime PCR

**Table 2:** Protein length and Genbank accession numbers of IRF4 in different species

Species	Protein length (aa)	GenBank No.
*Danio rerio*	460	NP_001116182
*Paralichthys olivaceus*	456	AEY55358
*Miichthys miiuy*	462	AHB59738
*Ictalurus punctatus*	433	AHH38752
*Oplegnathus fasciatus*	462	AFU81291
*Cynoglossus semilaevis*	457	XP_008332518
*Larimichthys crocea*	463	ATE88516
*Monopterus albus*	451	AFQ22942
*Salmo salar*	468	ACI33264
*Ophiophagus hannah*	437	ETE71355
*Gallus gallus*	445	AAK08199
*Mus musculus*	450	AAI37715
*Homo sapiens*	451	AAH15752
*Branchiostoma belcheri tsingtauense*	581	AJA02099

PCR products were analysed by electrophoresis on a 1% agarose gel and the anticipated fragments were purified from agarose gels. These fragments were ligated into the pMD18-T vector (TaKaRa) and transformed into competent *Escherichia. coli* DH-5α competent cells, and subsequently recombinants were identified and sequenced (Invitrogen).

Domains of deduced amino acid sequence search were performed with the simple modular architecture research tool (SMART) (http://smart.embl-heidelberg.de) and the conserved domain search program of NCBI (http://www.ncbi.nlm.nih.gov/Structure/cdd/wrpsb.cgi). Multiple sequence alignment was performed by the ClustalX program. Phylogenetic analysis was performed with ClustalX and the neighbor-joining methods with 1000 replicates of MEGA 5.0.

### Immune challenge *in vivo* and sample collection

For the challenge groups, 50 healthy common carp were intraperitoneally injected with 500 μl phosphate-buffered saline (PBS 7.4) containing polyinosinic:polycytudylic acid (poly I:C, 2.6 mg/ml, Sigma) or *Aeromonas hydrophila* (at a dose of 2.0 × 10^8^ cells). Before the experimental challenge in common carp, the bacteria were reactivated and cultured on Luria-Bertani (LB) medium in a shaker incubator at 28 °C overnight. The *A. hydrophila* was inactivated in 0.5% formalin at 37 °C for 36 h and then resuspended in PBS, followed by collection by centrifugation (3300 *g* for 10 min) and two washes with PBS. Whereas fish from the control group were injected with 500 μl of sterile PBS per fish.

The fish were anaesthetized with MS-222 (100 mg/L) and then sacrificed to collect experimental tissues. For both challenge experiments, three fish were sampled at 3, 6, 12, 24, 48 and 72 h post-injection from each group. Tissues were collected from each fish for total RNA extraction.

### Head kidney leukocytes (HKLs) isolation and immunity challenge *in vitro*

Common carp HKLs were prepared by Percoll (Sigma) gradients according to a previous report [38]. In short, head kidney tissue from freshly killed fish was passed through 100 μm stainless steel screens, and the resulting suspension was loaded onto freshly prepared 51/34% non-continuous Percoll density gradients and separated via centrifugation at 650 g for 30 min. After overnight recovery at 25 °C, 1 × 10^6^ cells were maintained in a 24-well tissue culture plate with poly I:C (500 μg/ml, SIGMA), LPS (1 mg/ml, SIGMA), peptidoglycan (PGN) (10 mg/ml, SIGMA), and flagellin (10 ng/ml, SIGMA).

### Construction of overexpression vectors

The full-length open reading frame (ORF) region of ccIRF4 was generated by PCR using Phusion HighFidelity DNA polymerase (PrimeSTAR) with the specific primers (Table 1) and then digested with the EcoRI/SacII restriction enzymes. Purified fragments ligated into the pcDNA3.1-EGFP vector (Invitrogen) and transformed into *E. coli* Top10 cells for adequate recombinant plasmid purification. The overexpression plasmid and vector-only pcDNA3.1-EGFP plasmid were extracted using an endotoxin-free plasmid isolation kit (TIANGEN) following manufacturer’s instructions. The extracted plasmids were dissolved in sterile ultrapure water, their concentrations were estimated by measuring the OD260, and they were then preserved at −20 °C for further analysis. The overexpression vector of ccMyD88 (abbreviation, pMyD88) was constructed using the same method and ligated into the fugw-2flag vector. The resulting overexpression vector pcDNA3.1-IRF4-EGFP (abbreviation, pIRF4) and pMyD88 were verified by sequencing.

### Transfection of EPC cells with pIRF4 overexpression plasmid

EPC cells were seeded in 24-well plates with 500 μL in each well at a concentration of 4 × 10^5^ cells/mL one day prior to transfections. Cells were transfected with the pIRF4 or empty vector once they reached about 80% confluency the following day with plasmids at 1 ug/well using X-tremeGENE HP DNA Transfection Reagent at 2 μl/well following the manufacturer’s instructions. EPC cells were collected after 48 h transfection with the pIRF4 or empty vector for quantification of associated immune molecule expressions (IFN, PKR, Viperin and IL-1β). The primers are listed in Table 1.

### RNA isolation and real-time PCR analysis

Total RNA was extracted from various tissues and EPC cells using an TRIzol reagent (TIANGEN). First-strand cDNA was synthesized using FastQuant RT kit (TIANGEN) in accordance with the manusfacture’s instructions. Real-time PCR was performed in a Rotor-Gene Q PCR instrument (Qiagen) with TransStart Tip Green qPCR SuperMix (Transgen). Real-time PCR conditions were 94 °C for 30 s, followed by 94 °C for 5 s, 60 °C for 30 s, and 70 °C 50 s for 40 cycles. Reactions were performed in 20 μl volume containing 10 μl SYBR green real-time PCR master mix, 6.8 μl double-distilled water, 0.6 μl of each primer, and 20 ng (2 μl) cDNA template. All samples were analyzed in triplicates and the expression value of all genes in common carp was calculated as relative to 40S ribosomal protein S11 gene or β-actin of EPC cells with the 2^(−∆∆C(T))^ method [42]. The primers were listed in Table 1.

### Luciferase activity assay

Dual-luciferase reporter assays to detect the effects of ccIRF4 on the activation of NF-κB were performed in 293 T cells by using pIRF4, pMyD88 or empty vector together with a luciferase-linked NF-κB. Transfection assays were performed using Lipofectamine 2000 (Invitrogen) according to the manufacturer’s instructions. The 293 T cells in 96-well plates were transfected with reporter gene plasmids, pGL-NF-κB-Luc, pGL-Renilla-luc plasmid (Promega), and the correct amount of expression plasmids or empty expression vectors (as control). The pGL-Renilla-luci plasmid was used as internal control. At 48 h post transfection, the Dual-Glo® Luciferase Reagent (Promega) was used to measure the activity of firefly and Renilla luciferase according to the manufacturer’s instructions with each experiment done in triplicates.

### Statistical analysis

Statistical analysis was performed using Graphpad Prism 6.0 software. The relative gene expression upon immune challenges was acquired using the 2^(−∆∆C(T))^ method. Data were expressed as the means standard deviation (SD) from at least three independent triplicated experiments. Significance differences were analysed using Student’s t-test for paired comparisons. Multiple comparison analysis was performed using one-way analysis of variance (ANOVA). All the data were homogeneous and normal, and *p* value of <0.05 was considered to be statistically significant.

## Results

### cDNA cloning and molecular characterization of the ccIRF4

The full-length cDNA of ccIRF4 was found to consist of 1885 bp. The ccIRF4 cDNA (GenBank accession No. OL365854) contains a 60 bp 5′-untranslated region (UTR), a 439 bp 3′-UTR containing mRNA instability motifs (^1857^AATAA^1863^), and an ORF of 1386 bp that translates into a 462 amino acid putative peptide with a predicted molecular mass of 52.3 kDa, the theoretical isoelectric was 6.105. The protein structure of ccIRF4 was predicted by SMART analysis. The deduced protein exhibited a DBD which possess five tryptophans (Trp21, Trp36, Trp48, Trp68, Trp87) and an IAD.

**Fig. 1:**
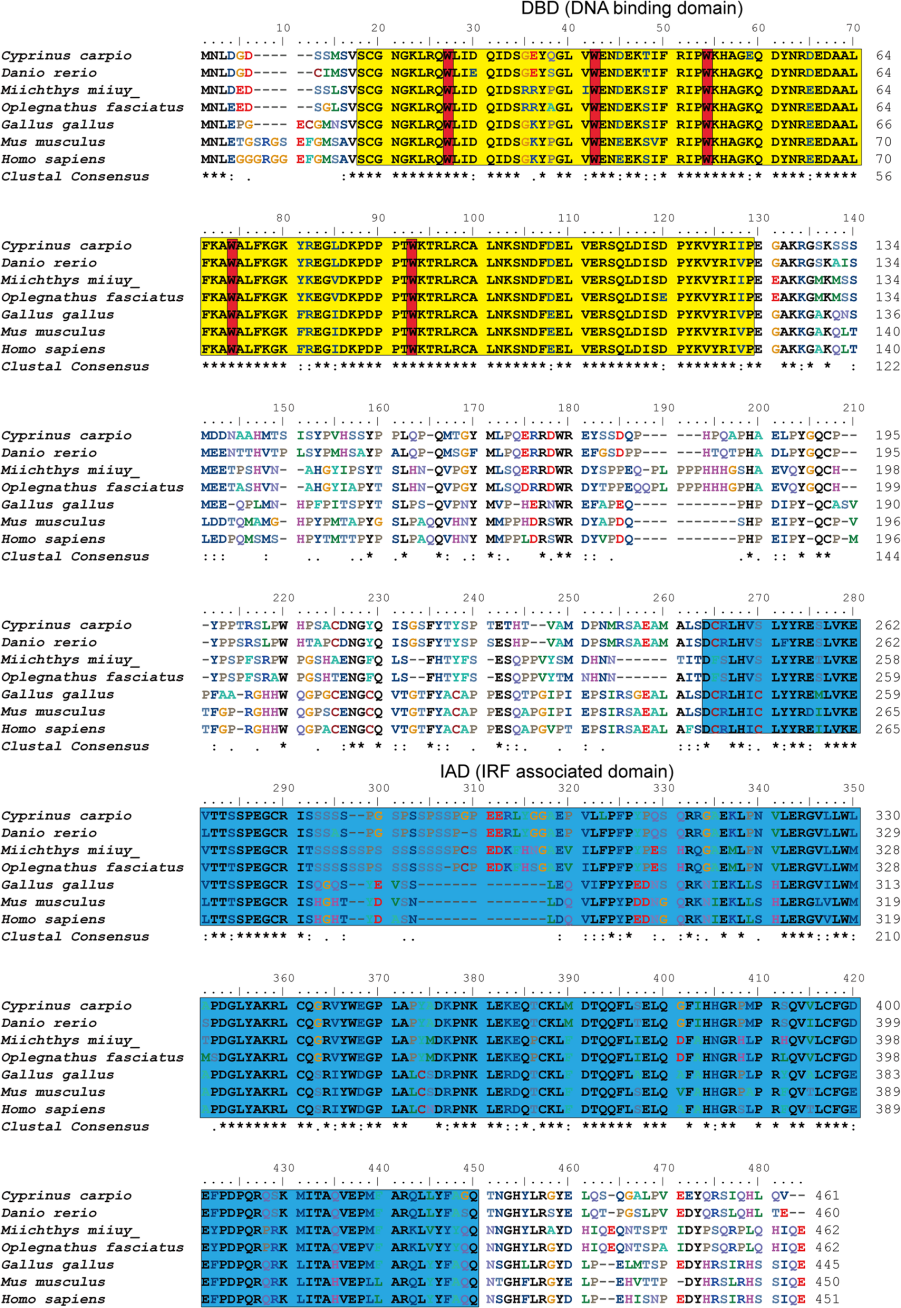
Multiple alignment IRF4 protein sequences in different species. The sequences were aligned using the ClustalW method. Identical (*) and similar (: or .) residues are indicated; the predicted domains from SMART Server software have been indicated by colored boxes: the yellow denotes the DBD and the blue denotes the IAD. Five tryptophan (W) residues are boxed in red. The GenBank accession numbers of the genes are listed in Table 2

**Fig. 2:**
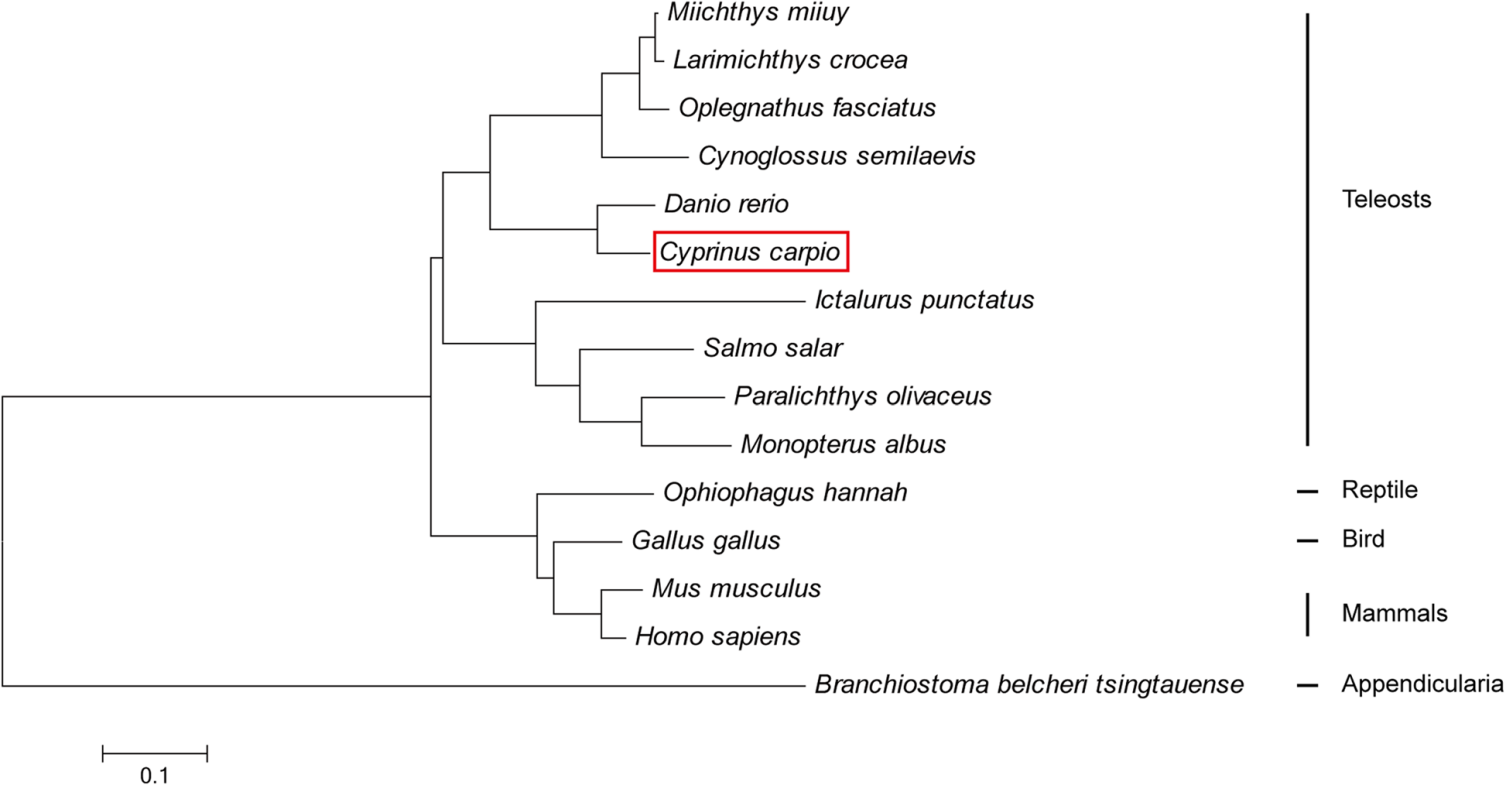
Phylogenetic analysis of IRF4 protein sequences from different species. The phylogenetic tree was produced by the neighbour-joining method in MEGA 5.0. The red frame shows the common carp IRF4

The multiple alignments of IRF4 between common carp and other species revealed conserved areas in all vertebrate groups. Significant homology was found in the putative DBD and IAD (Fig. 1). Furthermore, the phylogenetic tree including IRF4 sequences from all known species was constructed using the neighbor-joining method, which can be divided into several branches (teleosts, amphibia, birds, mammals and appendicularia). CcIRF4 had the closest relationship with zebrafish IRF4 (Fig. 2).

### Expression profile analysis of the ccIRF4 in healthy common carp

**Fig. 3:**
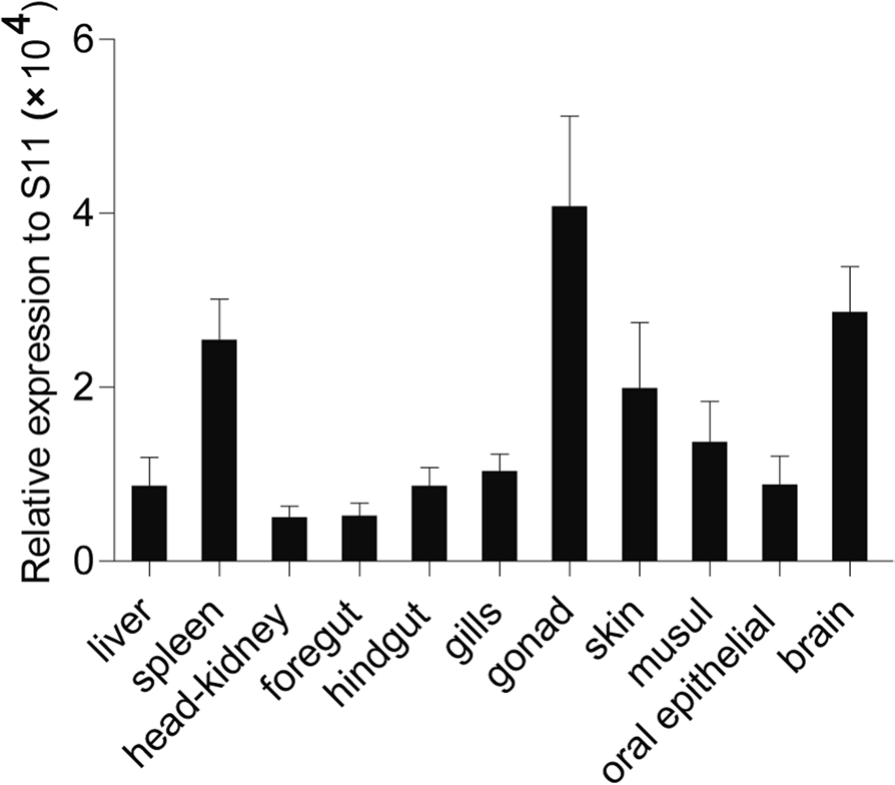
Tissue-specific expression of ccIRF4 under normal physiological conditions. CcIRF4 mRNA expression in the liver, spleen, head kidney, gills, skin, foregut, hindgut, buccal epithelium, gonad, muscle and brain were determined by real-time PCR. Gene expression levels were normalized using the 40S ribosomal protein S11 mRNA, *n* = 3

Real-time PCR was performed to examine the tissue distribution of ccIRF4 under normal physiological conditions in eleven tissues (liver, spleen, head-kidney, foregut, hindgut, gills, gonad, skin, muscle, oral epithelial and brain) of common carp. As a result, ccIRF4 mRNA expression was detected in all examined tissues, and broadly expressed in gonad, brain and spleen, moderately expressed in skin, muscle and gills, and weakly expressed in oral epithelial, hindgut, liver, foregut and head-kidney (Fig. 3).

### Expression profiles of ccIRF4 following poly I:C and *A. hydrophila* injection *in vivo*

**Fig. 4:**
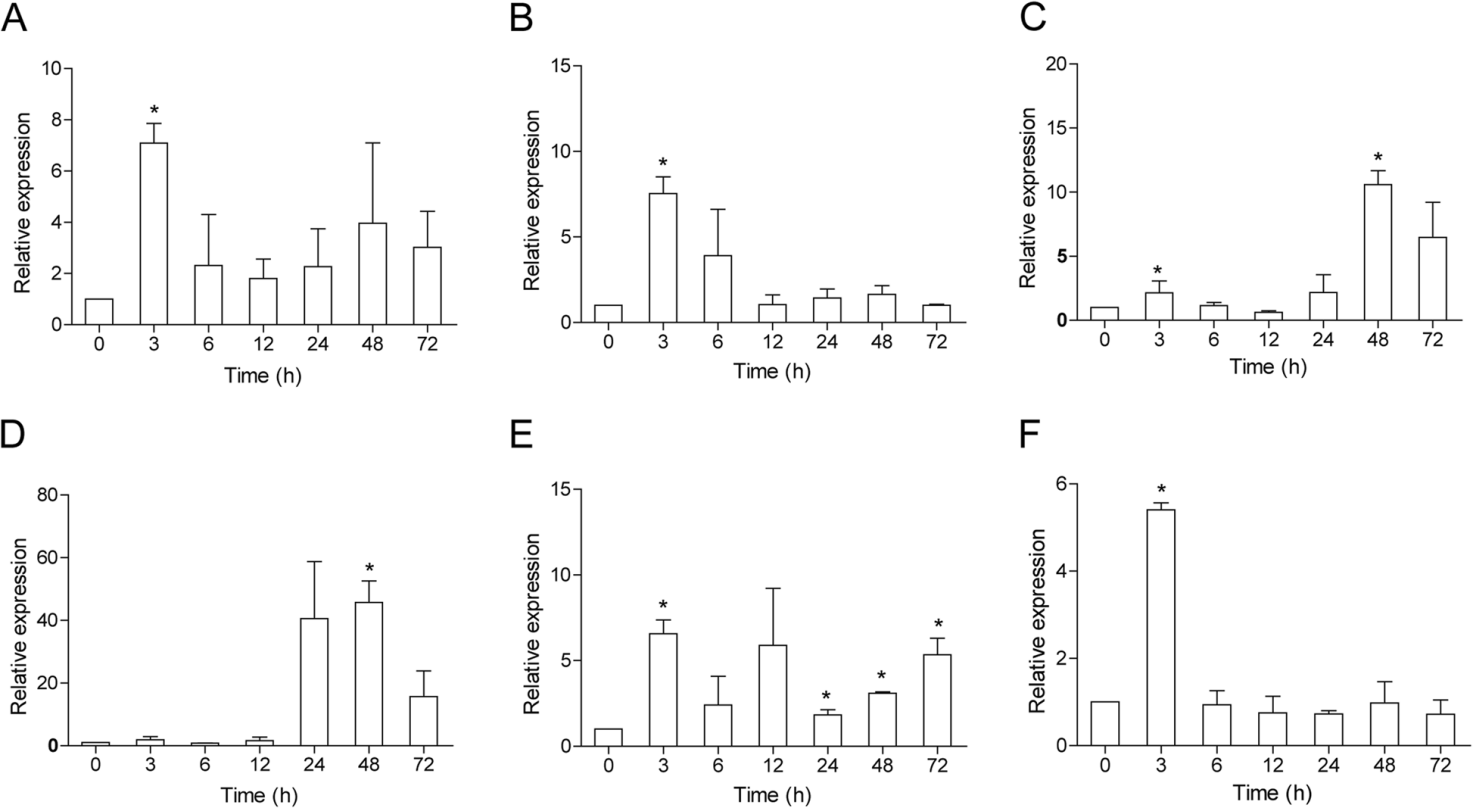
Expression analysis of ccIRF4 in response to Poly I:C challenge *in vivo*. Total RNA was extracted from the liver (**A**), spleen (**B**), head kidney (**C**), skin (**D**) foregut (**E**) and hindgut (**F**) at 0 (as control), 3, 6, 12, 24, 48 and 72 h post injection for real-time PCR. Expression was normalized using the 40S ribosomal protein S11 (*n* = 3, mean ± SD, *P < 0.05). The y-axis reveals fold change value relative to day 0, which has a different scale in Figure. 4 **A**-**F**

To investigate the role of ccIRF4 in immune response, the expression pattern of these genes in the immune-related tissues was examined after intraperitoneal injection with poly I:C and inactivated *A. hydrophila* at different time points. The expression profile of ccIRF4 after poly I:C injection is shown in Fig. 4. The peak expression of ccIRF4 appeared at 3 h post injection (hpi) in the liver, spleen, foregut and hindgut, with 7.0, 7.5-, 6.5- and 5.5-fold induction respectively. Meanwhile, the expression of ccIRF4 reached the highest level at 48 hpi in the head kidney (10.5-fold) and skin (45.7-fold).

**Fig. 5:**
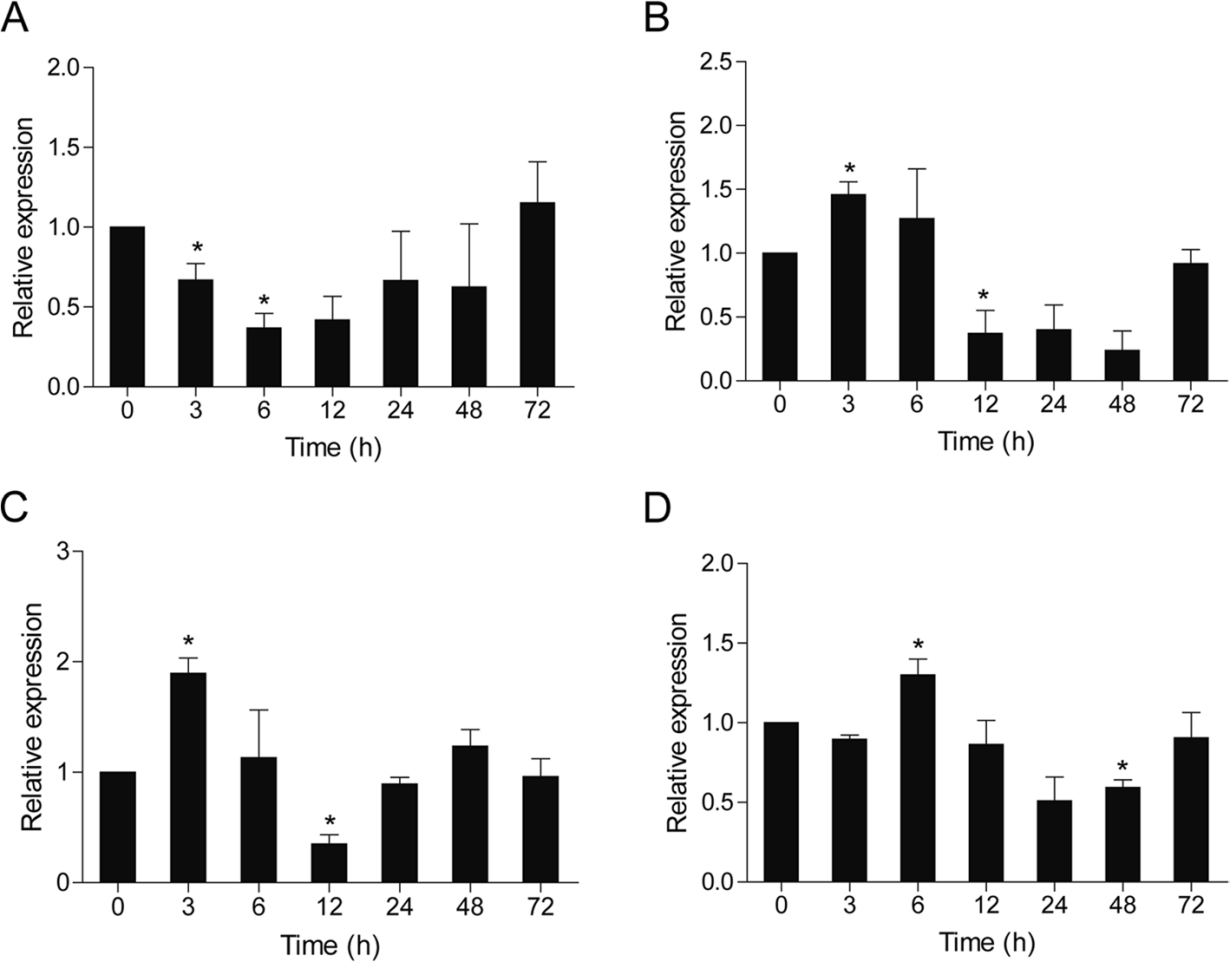
Expression analysis of ccIRF4 in response to *A. hydrophila* challenge *in vivo*. Total RNA was extracted from the spleen (**A**), head kidney (**B**), foregut (**C**) and hindgut (**D**) at 0 (as control), 3, 6, 12, 24, 48 and 72 h post injection for real-time PCR. Expression was normalized using the 40S ribosomal protein S11 (*n* = 3, mean ± SD, *P < 0.05)

The above results indicated that ccIRF4 might be involved in the antiviral immune response. Whether ccIRF4 participates in antibacterial immunity was also investigated. In *A. hydrophila* infected fish, expression of ccIRF4 was induced in the head kidney (1.4-fold) and foregut (1.8-fold) at 3 hpi and then down-regulated. In the hindgut, ccIRF4 exhibited the highest level at 6 hpi (1.3-fold). In contrast, the transcripts of ccIRF4 was reduced in the spleen (0.34-fold) (Fig. 5).

### Expression profiles of ccIRF4 upon immune stimulation in isolated HKLs

**Fig. 6:**
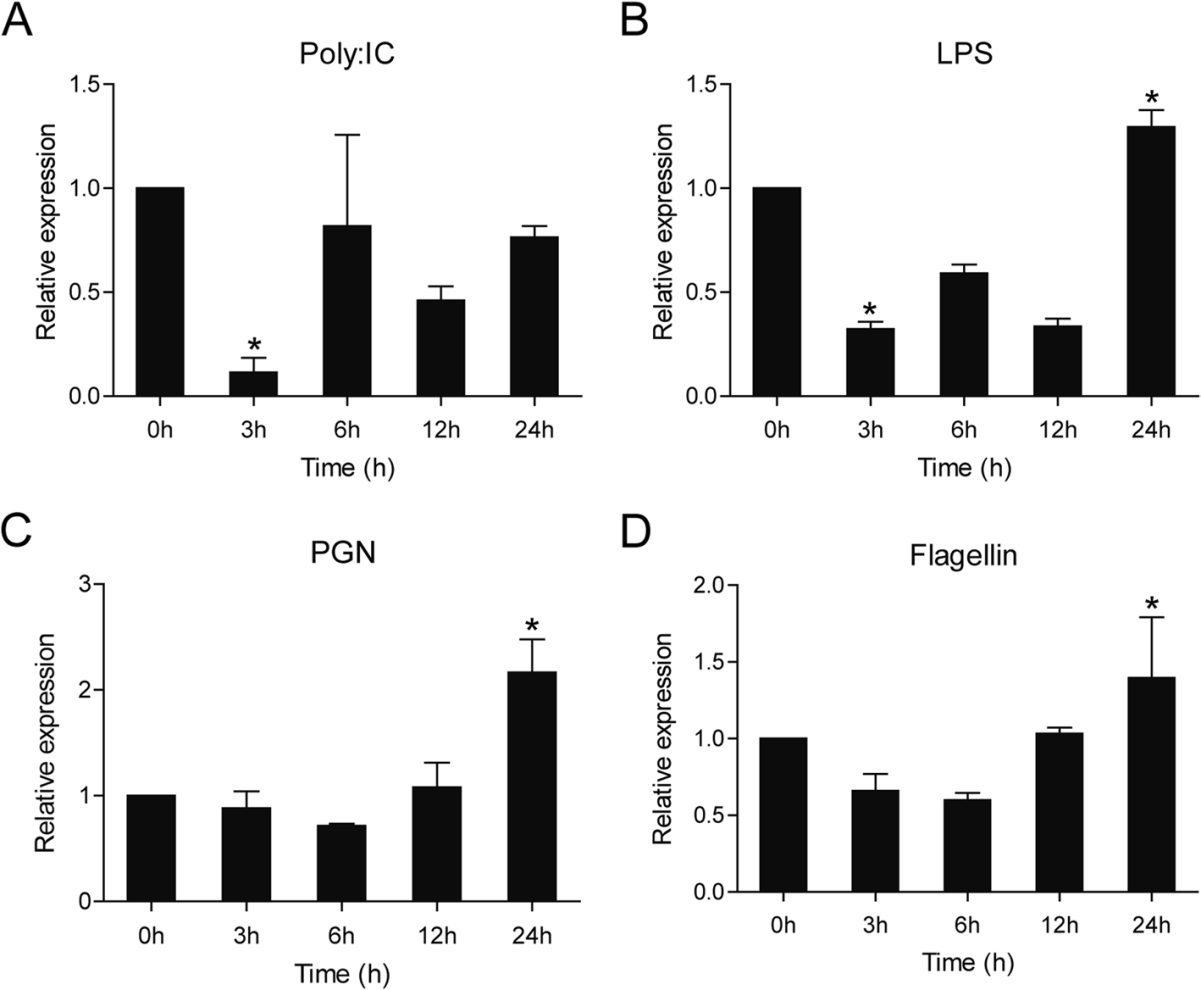
Expression levels of ccIRF4 in the HKLs upon different stimulation. The cells were collected at 0 (as control), 3, 6, 12 and 24 h post-infection for RNA extraction and real-time PCR analysis. Expression was normalized using the 40S ribosomal protein S11 (*n* = 3, mean ± SD, *P < 0.05)

Real-time PCR was used to examine the ccIRF4 transcription levels in isolated HKLs after stimulation with poly I:C, LPS, PGN and flagellin. As shown in Fig. 6, ccIRF4 expression was up-regulated by LPS (1.3-fold), PGN (2.1-fold), and flagellin (1.4-fold) at 24 h, but down-regulated by poly I:C with about 0.3-fold at 3 h.

### The mRNA expression of downstream IFN-associated factors in EPC cells overexpressing ccIRF4

**Fig. 7:**
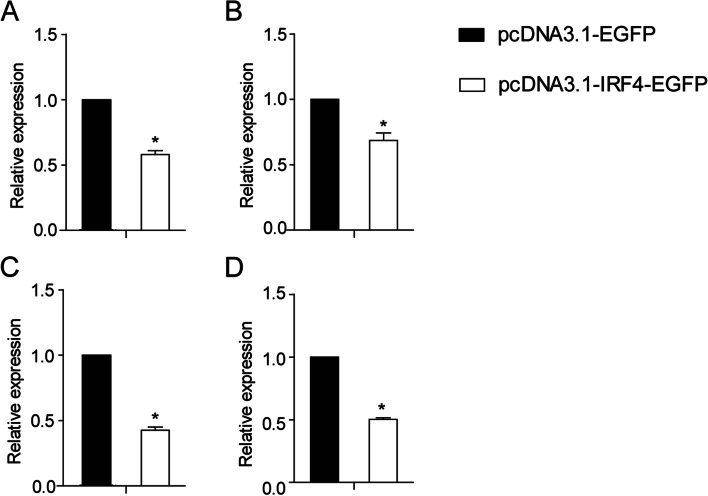
Relative expression of IFN (**A**), PKR (**B**), Viperin (**C**) and IL1-β (**D**) in ccIRF4-transfected EPC cells. Expression was normalized to β-actin (*n* = 3, mean ± SD, *P < 0.05)

Real-time PCR was used to detect the mRNA expression of IFN, PKR, Viperin and IL-1β genes after the empty vector and pIRF4 transfected EPC cells for 36 h. The result showed that the expression of IFN (0.58-fold, *P* < 0.05), PKR (0.69-fold, *P* < 0.05), Viperin (0.43-fold, *P* < 0.05), and IL-1β (0.51-fold, *P* < 0.05) genes were all down-regulated in EPC cells transfected with pIRF4 (Fig. 7).

### Regulation of NF-κB expession by ccIRF4

**Fig. 8:**
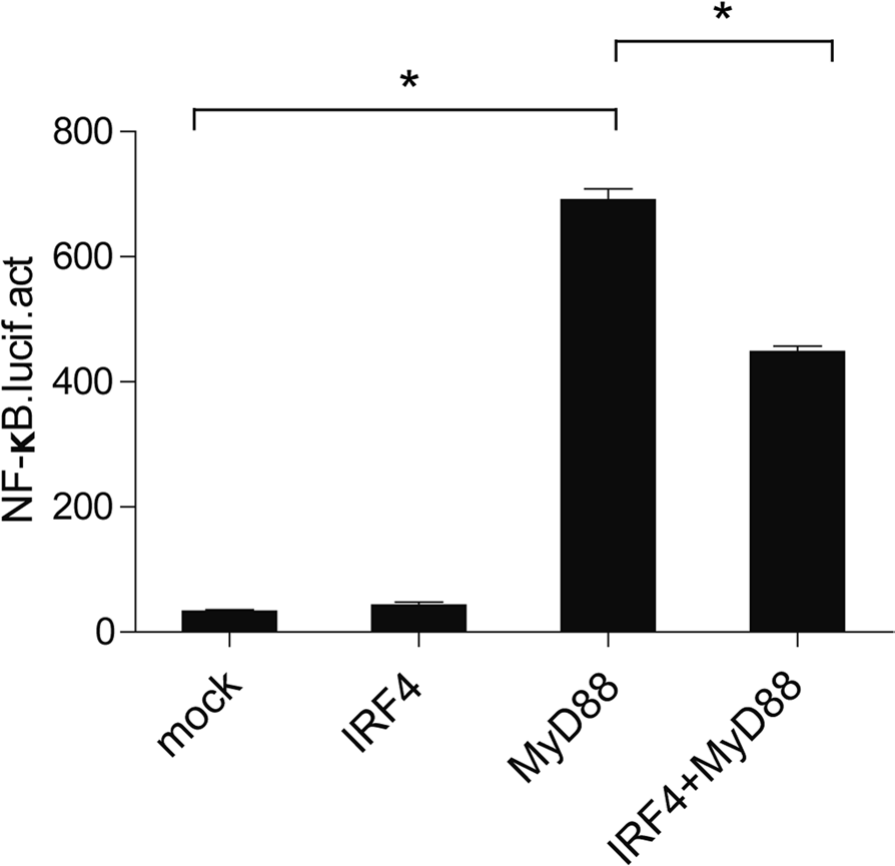
CcIRF4 negatively regulates the NF-κB pathway through MyD88. 293 T cells were cotransfected with the NF-κB reporter gene, together with ccIRF4 and MyD88 expression plasmids. Luciferase activity was measured after 48 h and determined against Renilla luciferase activity (mean ± SD, *P < 0.05)

To determine whether ccIRF4 could promote activation of NF-κB genes, dual-luciferase reporter assays were performed. Luciferase activity assays showed that NF-kB activity was not enhanced by overexpression of ccIRF4. While, NF-κB was significantly activated by overexpression of ccMyD88 (21.2-fold). However, co-transfection of ccIRF4 and ccMyd88 in 293 T cells inhibited activity of NF-κB compared to transfection of ccMyd88 alone (Fig. 8). These results suggest that ccIRF4 function as a transcription repressor in the NF-κB signalling pathway through ccMyD88.

## Discussion

IRF family plays an essential role in the host innate and adaptive immune responses in mammals and fish [43]. With the development of aquaculture, which is constantly challenged by outbreaks of infectious diseases inflicting considerable damages on this industry, increasing numbers of fish IRF genes are being identified and studied to investigate the immune system [44]. More recently, common carp IRF2 and IRF10 have been cloned [35, 40]. IRF4 has been extensively studied in mammalian species and is known to modulate anti-viral and anti-bacterial activity; however, information related to its functions in fish is very limited. In this study, IRF4 gene was cloned and its functional characteristics in immune response were investigated for the first time in common carp. Structural analysis revealed that ccIRF4 had two conserved functional domains, the N-terminus DBD and the C-terminus IAD. Multiple alignments of fish and vertebrate IRF4 sequences revealed a high degree of identity and the high sequence homology that exists in the DBD and IAD, suggesting that the functions of the IRF4 gene is likely to be conserved throughout the vertebrates. The DBD contains a five tryptophan repeat, which are highly conserved throughout the IRF family [6]. This region can form a helix-turn-helix motif and bind to the IFN stimulating response element (ISRE) and IRF regulatory element (IRF-E) in target promoters [45, 46]. In addition to the DBD, all IRFs (except IRF1 and IRF2) possess an IAD at the carboxyl-terminus, which is another conserved domain. This region is responsible for homo/hetero-dimers interactions of the IRFs and association with other transcription factors by the formation of transcriptional complexes [6]. These features are helpful for understanding the functions of IRF family in antiviral defense and immune regulation.

The phylogenetic tree of all known IRF4 amino acid sequences from different species was constructed using the neighbour-joining method. In the phylogenetic tree, IRF4s of teleosts, amphibia, birds and mammals were on a branch, while IRF4 of appendicularia was separate. Furthermore, ccIRF4 clustered with other fish IRF4 and demonstrated the closest phylogenetic relationship with that of zebrafish. The results match the established evolutionary relationships among the teleosts and other vertebrate species, supporting the authenticity of the nomenclature for the common carp IRF4 and suggest that ccIRF4 might exert similar functions as IRF4 in other fishes.

The ccIRF4 transcripts were detected in all the eleven tissues of healthy carps, indicating a constitutive transcription of IRF4. This ubiquitous tissue expression pattern supports previous studies of IRF4 in teleosts, including Asian swamp eel [23], rainbow trout [25], turbot [28], rock bream [29], half-smooth tongue sole [30], zebrafish [31] and blunt snout bream [34]. CcIRF4 appears to have higher transcript expression in spleen, an important tissue of the teleost immune system, which is a major site for the trapping and presentation of antigens for recognition by lymphocytes [47]. Surprisingly, the expression level of ccIRF4 in the gonad and brain was also high, implying that IRF4 may be involved in regulating the reproductive and nervous system of fish. However, ccIRF4 was expressed at very low levels in other tissues. Such expression pattern of ccIRF4 gene was similar to that in Atlantic cod, eel, chicken and mice, reflecting the possible similarity in protein function of IRF4 from different species [23, 47–49].

IRFs play a critical role in antiviral and antibacterial immunity. Previous studies in common carp showed that expression of IRF1, IRF2, IRF3, IRF5, IRF7, IRF9 and IRF10 were up-regulated upon stimulation with poly I:C, viruses and/or bacteria [35–40]. Spleen and head kidney were selected to perform the study, as these organs represent lymphoid and myeloid tissues, which are important in fish immune system. We also performed the study in the foregut and hindgut in order to determine whether ccIRF4 participate in regulating the mucosal immune system. Poly I:C, a well-established inducer of fish type I IFNs and ISGs, is a synthetic mimic of dsRNA which recognized by TLR3 and TLR22 [50, 51]. Similarly with the expression profile in eel and large yellow croaker [23, 27], in our present study, ccIRF4 expression was enhanced after stimulation with poly I: C in all the six tissues. The maximum induction of ccIRF4 in liver, spleen, foregut and hindgut occurred at 3 hpi, earlier than that in head kidney and skin (at 48 hpi), maybe because those organs are the first sites against the invading antiviral pathogens. The induction in the skin (45.7-fold) was much stronger than that in the other tissues (5.5- to 10.5-fold), revealing the important role of ccIRF4 in the mucosal immune system response to poly I:C. Besides, the expression of rock bream IRF4 was induced in the head kidney post-injection with poly I:C and rock bream iridovirus [29]. Furthermore, two isoforms, IRF4a and IRF4b, have been identified in half-smooth tongue sole, and both of them were up-regulated upon megalocytivirus infection in the spleen, head kidney and liver [29]. However, poly I:C has no impact on the expression of human IRF4, indicating the different role of IRF4 in human and fish [21].

It should be noted that *A. hydrophila* (a well-known fish bacterial pathogen) infection up-regulated the expression of ccIRF4 in the head kidney, foregut and hindgut, but its expression in the spleen was down-regulated. This is inconsistent with the studies that *Vibro harveyi* and *Edwardsiella Tarda* down-regulated the expression of half-smooth tongue sole IRF4a [30] and LPS down-regulated the expression of rock bream and rainbow trout IRF4 [25, 29]. Eel IRF4 had whereas relatively lower fold change induced by LPS and *A. hydrophila* [23]. Besides, half-smooth tongue sole IRF4b was up-regulated after bacterial infection [30]. These results suggest that IRF4 in different species may respond to different pathogens. However, further studies should be conducted to investigate the mechanism of its regulatory role during the bacterial and viral infection.

HKLs consist of heterogeneous cells and are widely used as an experimental system to study immune responses [52, 53]. Thus, we isolated the leukocytes from head kidney for further investigations to gain a better understanding of the antimicrobial mechanisms of ccIRF4. According to the observed expression level of ccIRF4, which was increased after stimulation with different ligands (LPS, PGN and Flagellin) in HKLs, it could be further confirmed that ccIRF4 may play substantial roles to protect the host from bacteria. However, ccIRF4 in HKLs was down regulated by poly I:C. IRF4 gene expression in channel catfish was found to be up-regulated by poly I:C in a mixed macrophage/T cell culture and down regulated in B cells, indicating whether poly I:C can induce IRF4 expression depends on the cell types [54]. What’s more, IRF4 is expressed at low levels in early B cell development stages and markedly up-regulated in later stages. All the above may explain the low sensitivity of ccIRF4 to poly I:C treatment in HKLs, which are consist of T cells, B cells, granulocytes, monocytes and macrophage. Even so, it could be suggested that ccIRF4 participate in both antibacterial and antiviral innate immunity.

To date, very few studies on the regulatory roles of the IRF4 subfamily members in the IFN or NF-κB signalling pathway have appeared in the literature. In the current study, we detected the mRNA expression of Type I IFN, ISGs (PKR, Viperin), and IL-1β in transfected EPC cells. Zebrafish IRF4 induced the IFN promoter activity [31]. On the contrary, our results showed that overexpression of ccIRF4 in EPC cells down-regulated the production of type I IFN, PKR, Viperin and IL-1β. In mammals, IRF4 possesses the ability to negatively regulate TLR signalling pathway by competing with IRF5 for binding to Myd88, which is a signalling adaptor molecule [19]. In the present study, dual luciferase reporter assays showed that ccIRF4 inhibited the NF-κB signal pathway mediated by MyD88. The results demonstrated that ccIRF4 has a similar function to that of mammalian IRF4, which can negatively regulate the TLR signalling pathway. Whereas, its regulatory role in the IFN system is not conserved in fish.

## Conclusions

In summary, we have identified and characterized the ccIRF4 from common carp. The mRNA expression profile showed that ccIRF4 was expressed in all the eleven tissues. In addition, ccIRF4 was found to participate in the antiviral and antibacterial immunity both *in vivo* and *in vitro*. Moreover, ccIRF4 has been identified as a negative regulator in both of the IFN and NF-κB signalling pathways. The results obtained in this study provide a basis for further, more detailed investigations into the functions of fish IRF4.

## Data Availability

The dataset supporting the conclusions of this article is available in the GenBank (https://www.ncbi.nlm.nih.gov/nuccore/OL365854) and the accession number is OL365854.

## Abbreviations

IFN: Interferon
IRF: Interferon regulatory factor;
DBD: DNA binding domain
IAD: IRF-associated domain
poly (I:C): polyinosinic:polycytidylic acid
LPS: Lipopolysaccharide
PGN: Peptidoglycan
ISG: IFN-stimulated gene
ISRE: IFN stimulated response element
IL: Interleukin
TLR: Toll-like receptor
PAMP: Pathogen-associated molecular pattern
EPC: Epithelioma papulosum cyprinid
FBS: Fetal bovine serum
RACE: Rapid amplification of the cDNA ends
SMART: Simple modular architecture research tool
PBS: Phosphate-buffered saline
LB: Luria-Bertani
HKL: Head kidney leukocyte
ORF: Open reading frame
ANOVA: One-way analysis of variance
Trp: Tryptophans
NJ: Neighbor-joining
hpi: Hour post injection
ISRE: IFN stimulating response element
IRF-E: IRF regulatory element

## References

[1] Su J, Heng J, Huang T, Peng L, Yang C, Li Q (2012). Identification, mRNA expression and genomic structure of TLR22 and its association with GCRV susceptibility/resistance in grass carp (Ctenopharyngodon idella). Dev Comp Immunol.

[2] Barnes B, Lubyova B, Pitha PM (2002). On the role of IRF in host defense. J Interf Cytokine Res.

[3] Savitsky D, Tamura T, Yanai H, Taniguchi T (2010). Regulation of immunity and oncogenesis by the IRF transcription factor family. Cancer Immunol Immunother.

[4] Paun A, Pitha PM (2007). The IRF family, revisited. Biochimie..

[5] Stellacci E, Testa U, Petrucci E, Benedetti E, Orsatti R, Feccia T, Stafsnes M, Coccia EM, Marziali G, Battistini A (2004). Interferon regulatory factor-2 drives megakaryocytic differentiation. Biochem J.

[6] Eroshkin A, Mushegian A (1999). Conserved transactivation domain shared by interferon regulatory factors and Smad morphogens. J Mol Med (Berl).

[7] Tailor P, Tamura T, Ozato K (2006). IRF family proteins and type I interferon induction in dendritic cells. Cell Res.

[8] Zhang R, Chen K, Peng L, Xiong H (2012). Regulation of T helper cell differentiation by interferon regulatory factor family members. Immunol Res.

[9] Shukla V, Lu R (2014). IRF4 and IRF8: Governing the virtues of B Lymphocytes. Front Biol (Beijing).

[10] Finlay D (2014). IRF4 links antigen affinity to CD8+ T-cell metabolism. Immunol Cell Biol.

[11] Hu CM, Jang SY, Fanzo JC, Pernis AB (2002). Modulation of T cell cytokine production by interferon regulatory factor-4. J Biol Chem.

[12] Lu R (2008). Interferon regulatory factor 4 and 8 in B-cell development. Trends Immunol.

[13] Sciammas R, Shaffer AL, Schatz JH, Zhao H, Staudt LM, Singh H (2006). Graded expression of interferon regulatory factor-4 coordinates isotype switching with plasma cell differentiation. Immunity..

[14] Rengarajan J, Mowen KA, McBride KD, Smith ED, Singh H, Glimcher LH (2002). Interferon regulatory factor 4 (IRF4) interacts with NFATc2 to modulate interleukin 4 gene expression. J Exp Med.

[15] Lee CH, Melchers M, Wang H, Torrey TA, Slota R, Qi CF, Kim JY, Lugar P, Kong HJ, Farrington L (2006). Regulation of the germinal center gene program by interferon (IFN) regulatory factor 8/IFN consensus sequence-binding protein. J Exp Med.

[16] Honma K, Udono H, Kohno T, Yamamoto K, Ogawa A, Takemori T, Kumatori A, Suzuki S, Matsuyama T, Yui K (2005). Interferon regulatory factor 4 negatively regulates the production of proinflammatory cytokines by macrophages in response to LPS. Proc Natl Acad Sci U S A.

[17] Brustle A, Heink S, Huber M, Rosenplanter C, Stadelmann C, Yu P, Arpaia E, Mak TW, Kamradt T, Lohoff M (2007). The development of inflammatory T(H)-17 cells requires interferon-regulatory factor 4. Nat Immunol.

[18] Huber M, Brustle A, Reinhard K, Guralnik A, Walter G, Mahiny A, von Low E, Lohoff M (2008). IRF4 is essential for IL-21-mediated induction, amplification, and stabilization of the Th17 phenotype. Proc Natl Acad Sci U S A.

[19] Negishi H, Ohba Y, Yanai H, Takaoka A, Honma K, Yui K, Matsuyama T, Taniguchi T, Honda K (2005). Negative regulation of Toll-like-receptor signaling by IRF-4. Proc Natl Acad Sci U S A.

[20] Xu D, Meyer F, Ehlers E, Blasnitz L, Zhang L (2011). Interferon regulatory factor 4 (IRF-4) targets IRF-5 to regulate Epstein-Barr virus transformation. J Biol Chem.

[21] Demoulins T, Baron ML, Kettaf N, Abdallah A, Sharif-Askari E, Sekaly RP (2009). Poly (I:C) induced immune response in lymphoid tissues involves three sequential waves of type I IFN expression. Virology..

[22] Laghari ZA, Li L, Chen SN, Huo HJ, Huang B, Zhou Y, Nie P (2018). Composition and transcription of all interferon regulatory factors (IRFs), IRF111 in a perciform fish, the mandarin fish, Siniperca chuatsi. Dev Comp Immunol.

[23] Xu QQ, Yang DQ, Tuo R, Wan J, Chang MX, Nie P (2014). Gene cloning and induced expression pattern of IRF4 and IRF10 in the Asian swamp eel (Monopterus albus). Dongwuxue Yanjiu.

[24] Lai CF, Wang TY, Yeh MI, Chen TY (2020). Characterization of orange-spotted grouper (Epinephelus coioides) interferon regulatory factor 4 regulated by heat shock factor 1 during heat stress in response to antiviral immunity. Fish Shellfish Immunol..

[25] Holland JW, Karim A, Wang T, Alnabulsi A, Scott J, Collet B, Mughal MS, Secombes CJ, Bird S (2010). Molecular cloning and characterization of interferon regulatory factors 4 and 8 (IRF-4 and IRF-8) in rainbow trout, Oncorhynchus mykiss. Fish Shellfish Immunol.

[26] Liu D, Chen J, Zhang H, Hu M, Lou H, Liu Q, Zhang S, Hu G (2016). Interferon regulatory factor 4b (IRF4b) in Japanese flounder, Paralichthys olivaceus: Sequencing, ubiquitous tissue distribution and inducible expression by poly(I:C) and DNA virus. Dev Comp Immunol.

[27] Tang J, Jiang L, Liu W, Lou B, Wu C, Zhang J (2018). Expression and functional characterization of interferon regulatory factors 4, 8, and 9 in large yellow croaker (Larimichthys crocea). Dev Comp Immunol.

[28] Li S, Hu G, Chen Z, Song L, Wang G, Liu D, Liu Q (2018). Cloning and expression study of an IRF4a gene and its two transcript variants in turbot, Scophthalmus maximus. Fish Shellfish Immunol.

[29] Bathige SD, Whang I, Umasuthan N, Lim BS, Park MA, Kim E, Park HC, Lee J (2012). Interferon regulatory factors 4 and 8 in rock bream, Oplegnathus fasciatus: structural and expressional evidence for their antimicrobial role in teleosts. Fish Shellfish Immunol..

[30] Zhang J, Li YX, Hu YH (2015). Molecular characterization and expression analysis of eleven interferon regulatory factors in half-smooth tongue sole, Cynoglossus semilaevis. Fish Shellfish Immunol.

[31] Ai K, Luo K, Li Y, Hu W, Gao W, Fang L, Tian G, Ruan G, Xu Q (2017). Expression pattern analysis of IRF4 and its related genes revealed the functional differentiation of IRF4 paralogues in teleost. Fish Shellfish Immunol..

[32] Leong JS, Jantzen SG, von Schalburg KR, Cooper GA, Messmer AM, Liao NY, Munro S, Moore R, Holt RA, Jones SJ (2010). Salmo salar and Esox lucius full-length cDNA sequences reveal changes in evolutionary pressures on a post-tetraploidization genome. BMC Genomics.

[33] Liu S, Zhang Y, Zhou Z, Waldbieser G, Sun F, Lu J, Zhang J, Jiang Y, Zhang H, Wang X (2012). Efficient assembly and annotation of the transcriptome of catfish by RNA-Seq analysis of a doubled haploid homozygote. BMC Genomics.

[34] Zhan FB, Jakovlic I, Wang WM (2019). Identification, characterization and expression in response to Aeromonas hydrophila challenge of five interferon regulatory factors in Megalobrama amblycephala. Fish Shellfish Immunol..

[35] Li H, Chen X, Zhu Y, Liu R, Zheng L, Shan S, Zhang F, An L, Yang G (2021). Molecular characterization and immune functional analysis of IRF2 in common carp (Cyprinus carpio L.): different regulatory role in the IFN and NF-kappaB signalling pathway. BMC Vet Res.

[36] Shan S, Qi C, Zhu Y, Li H, An L, Yang G (2016). Expression profile of carp IFN correlate with the up-regulation of interferon regulatory factor-1 (IRF-1) in vivo and in vitro: the pivotal molecules in antiviral defense. Fish Shellfish Immunol..

[37] Feng H, Liu H, Kong R, Wang L, Wang Y, Hu W, Guo Q (2011). Expression profiles of carp IRF-3/−7 correlate with the up-regulation of RIG-I/MAVS/TRAF3/TBK1, four pivotal molecules in RIG-I signaling pathway. Fish Shellfish Immunol.

[38] Zhu Y, Qi C, Shan S, Zhang F, Li H, An L, Yang G (2016). Characterization of common carp (Cyprinus carpio L.) interferon regulatory factor 5 (IRF5) and its expression in response to viral and bacterial challenges. BMC Vet Res.

[39] Zhu Y, Shan S, Feng H, Jiang L, An L, Yang G, Li H (2019). Molecular characterization and functional analysis of interferon regulatory factor 9 (irf9) in common carp Cyprinus carpio: a pivotal molecule in the Ifn response against pathogens. J Fish Biol.

[40] Zhu Y, Shan S, Zhao H, Liu R, Wang H, Chen X, Yang G, Li H (2020). Identification of an IRF10 gene in common carp (Cyprinus carpio L.) and analysis of its function in the antiviral and antibacterial immune response. BMC Vet Res.

[41] Sun F, Zhang YB, Liu TK, Gan L, Yu FF, Liu Y, Gui JF (2010). Characterization of fish IRF3 as an IFN-inducible protein reveals evolving regulation of IFN response in vertebrates. J Immunol.

[42] Livak KJ, Schmittgen TD (2001). Analysis of relative gene expression data using real-time quantitative PCR and the 2(−Delta Delta C(T)) Method. Methods..

[43] Zhang YB, Gui JF (2012). Molecular regulation of interferon antiviral response in fish. Dev Comp Immunol.

[44] Lafferty KD, Harvell CD, Conrad JM, Friedman CS, Kent ML, Kuris AM, Powell EN, Rondeau D, Saksida SM (2015). Infectious diseases affect marine fisheries and aquaculture economics. Annu Rev Mar Sci.

[45] Honda K, Taniguchi T (2006). IRFs: master regulators of signalling by Toll-like receptors and cytosolic pattern-recognition receptors. Nat Rev Immunol.

[46] Escalante CR, Yie J, Thanos D, Aggarwal AK (1998). Structure of IRF-1 with bound DNA reveals determinants of interferon regulation. Nature..

[47] Inkpen SM, Hori TS, Gamperl AK, Nash GW, Rise ML (2015). Characterization and expression analyses of five interferon regulatory factor transcripts (Irf4a, Irf4b, Irf7, Irf8, Irf10) in Atlantic cod (Gadus morhua). Fish Shellfish Immunol..

[48] Dougherty DC, Park HM, Sanders MM (2009). Interferon regulatory factors (IRFs) repress transcription of the chicken ovalbumin gene. Gene..

[49] Takaoka A, Tamura T, Taniguchi T (2008). Interferon regulatory factor family of transcription factors and regulation of oncogenesis. Cancer Sci.

[50] Matsuo A, Oshiumi H, Tsujita T, Mitani H, Kasai H, Yoshimizu M, Matsumoto M, Seya T (2008). Teleost TLR22 recognizes RNA duplex to induce IFN and protect cells from birnaviruses. J Immunol.

[51] Bergan V, Steinsvik S, Xu H, Kileng O, Robertsen B (2006). Promoters of type I interferon genes from Atlantic salmon contain two main regulatory regions. FEBS J.

[52] Zhang F, Liu D, Wang L, Li T, Chang Q, An L, Yang G (2015). Characterization of IgM-binding protein: A pIgR-like molecule expressed by intestinal epithelial cells in the common carp (Cyprinus carpio L.). Vet Immunol Immunopathol.

[53] Li H, Li T, Guo Y, Li Y, Zhang Y, Teng N, Zhang F, Yang G (2018). Molecular characterization and expression patterns of a non-mammalian toll-like receptor gene (TLR21) in larvae ontogeny of common carp (Cyprinus carpio L.) and upon immune stimulation. BMC Vet Res.

[54] Milev-Milovanovic I, Majji S, Thodima V, Deng Y, Hanson L, Arnizaut A, Waldbieser G, Chinchar VG (2009). Identification and expression analyses of poly [I:C]-stimulated genes in channel catfish (Ictalurus punctatus). Fish Shellfish Immunol.

